# Fracture stability modifies the risk profile and outcomes of proximal femoral nailing versus hemiarthroplasty for elderly intertrochanteric fractures

**DOI:** 10.1186/s12891-025-09471-5

**Published:** 2026-01-14

**Authors:** Yusheng Zhang, Caizhen Luo, Wei Chen, Wanfei Wu, Huanyu Shi, Dongchu Zhao, Yan Xiong, Yang Li

**Affiliations:** 1 Department of Emergency Medicine, Medical Center of Trauma and War Injury, Daping Hospital, Army Medical University, State Key Laboratory of Trauma and Chemical Poisoning, Chongqing, 400042 China; 2 Medical Center of Trauma and War Injury, Daping Hospital, Army Medical University, Chongqing, 400042 China; 3 Department of Orthopedics, Daping Hospital, Army Medical University, No. 10, Daping Changjiang Branch Road, Yuzhong District, Chongqing, 400042 China; 4 Department of Emergency Medicine, Medical Center of Trauma and War Injury, Daping Hospital, Army Medical University, State Key Laboratory of Trauma and Chemical Poisoning, No. 10, Daping Changjiang Branch Road, Yuzhong District, Chongqing, 400042 China

**Keywords:** Intertrochanteric fracture, Hemiarthroplasty, Proximal femoral nail antirotation, Fracture stability, Trade-off

## Abstract

**Background:**

With population aging, intertrochanteric fractures (ITFs) in elderly individuals are increasingly common, and the choice between proximal femoral nail antirotation (PFNA) and hemiarthroplasty (HA) remains under debate, particularly regarding the optimal balance between early mobilization and the risk of long-term implant failure or revision. This study aimed to investigate this trade-off through a retrospective cohort comparison of PFNA and HA in elderly ITF patients stratified by fracture stability.

**Methods:**

This retrospective cohort study reviewed 372 elderly patients with ITF treated from 2016 to 2022. Patients were grouped by surgery (PFNA or HA) and stratified by fracture stability (AO/OTA classification). The primary outcome was the Harris hip score (HHS) at 12 months post-operatively; secondary outcomes included perioperative metrics and complications.

**Results:**

Overall, PFNA group demonstrated higher 12-month HHS (87.9 ± 5.8 vs. 84.6 ± 6.3, *p* < 0.001) despite longer operative duration, hospital stays, and delayed weight-bearing (*p* < 0.05) than HA. Subgroup analysis revealed no difference in HHS at 12 months for stable ITF patients. In the unstable subgroup, HA enabled earlier weight-bearing (4.5 ± 3.3 vs. 8.5 ± 3.7 days, *p* < 0.001), but PFNA demonstrated significantly better 12-month HHS (88.3 ± 6.3 vs. 82.5 ± 6.3, *p* < 0.001), with less blood loss and lower costs.

**Conclusions:**

For stable ITFs in elderly patients, the PFNA and HA yield comparable outcomes. For unstable ITFs, a clinical trade-off exists: HA provides faster early mobilization, whereas PFNA offers superior long-term hip function. Implant choice should be tailored to patient life expectancy and functional goals.

## Introduction

With rapid population aging [1], China has the largest population of elderly people with hip fractures in the world [2], with studies reporting an intertrochanteric fracture (ITF) incidence rate in urban areas of approximately 1800 per million individuals [3]. The average surgical intervention rate for elderly patients with hip fractures in China is 59.6%, with surgery being the definitive therapeutic option [4]. Crucially, for these geriatric patients, surgery is recommended as promptly as systemic conditions allow, as delays beyond the optimal 48-hour window can increase mortality and complication rates, such as deep vein thrombosis [5, 6]. Furthermore, recent global challenges, such as the COVID-19 pandemic, have underscored the critical need for rapid surgical decision-making and the paramount importance of pre-existing patient comorbidities in determining perioperative risk and outcomes for this vulnerable patient cohort [7]. Proximal femoral nail antirotation (PFNA) and hemiarthroplasty (HA) are two commonly employed surgical strategies for treating ITF in elderly patients [8]. The decision between PFNA and HA is frequently guided by the orthopedic surgeon’s area of expertise; trauma orthopedists typically prefer PFNA, whereas joint surgeons exhibit greater skill in performing HA. However, robust evidence from real-world clinical practice remains limited, particularly concerning how fracture stability should influence the choice between these two common procedures.

The objective of our study was to fill the existing knowledge gap by directly comparing the clinical outcomes of PFNA and HA for both stable and unstable ITF in elderly individuals. Despite numerous comparative studies, few studies have explored the dynamic trade-off between short-term recovery speed and long-term functional prognosis in unstable fracture subtypes. This study aims to elucidate this trade-off through a large-sample retrospective analysis, providing evidence for personalized treatment of unstable intertrochanteric fractures in elderly individuals. Using a retrospective cohort study methodology, we systematically analyzed perioperative metrics, postoperative recovery, and long-term outcomes. Our goal is to contribute valuable insights to the ongoing discourse on the optimal management of the ITF in elderly individuals.

## Methods

### Study design and patient selection

**Fig. 1:**
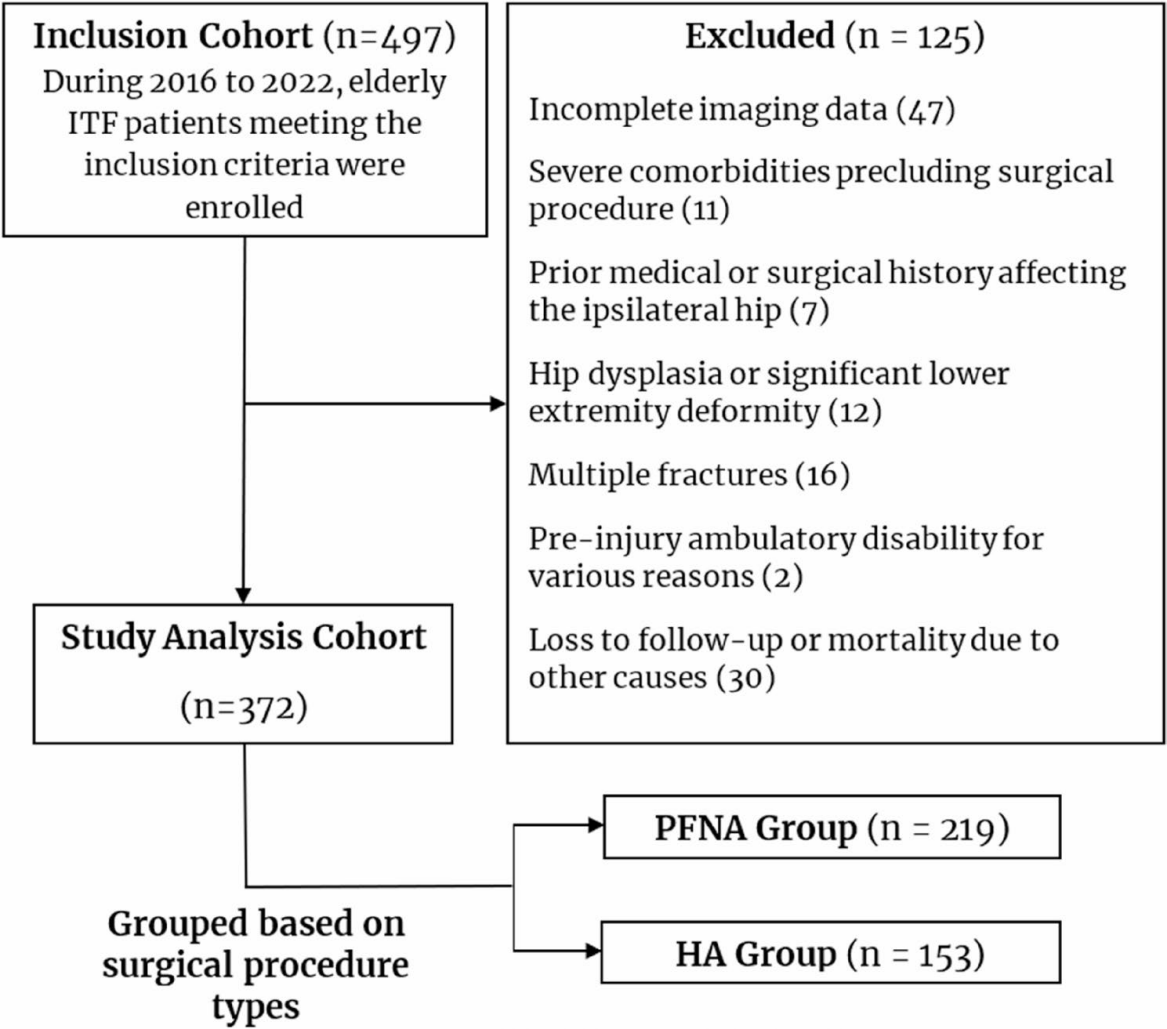
Flow chart of ITF patient screening. ITF: intertrochanteric fracture; PFNA: proximal femoral nail antirotation; HA: hemiarthroplasty

**Fig. 2:**
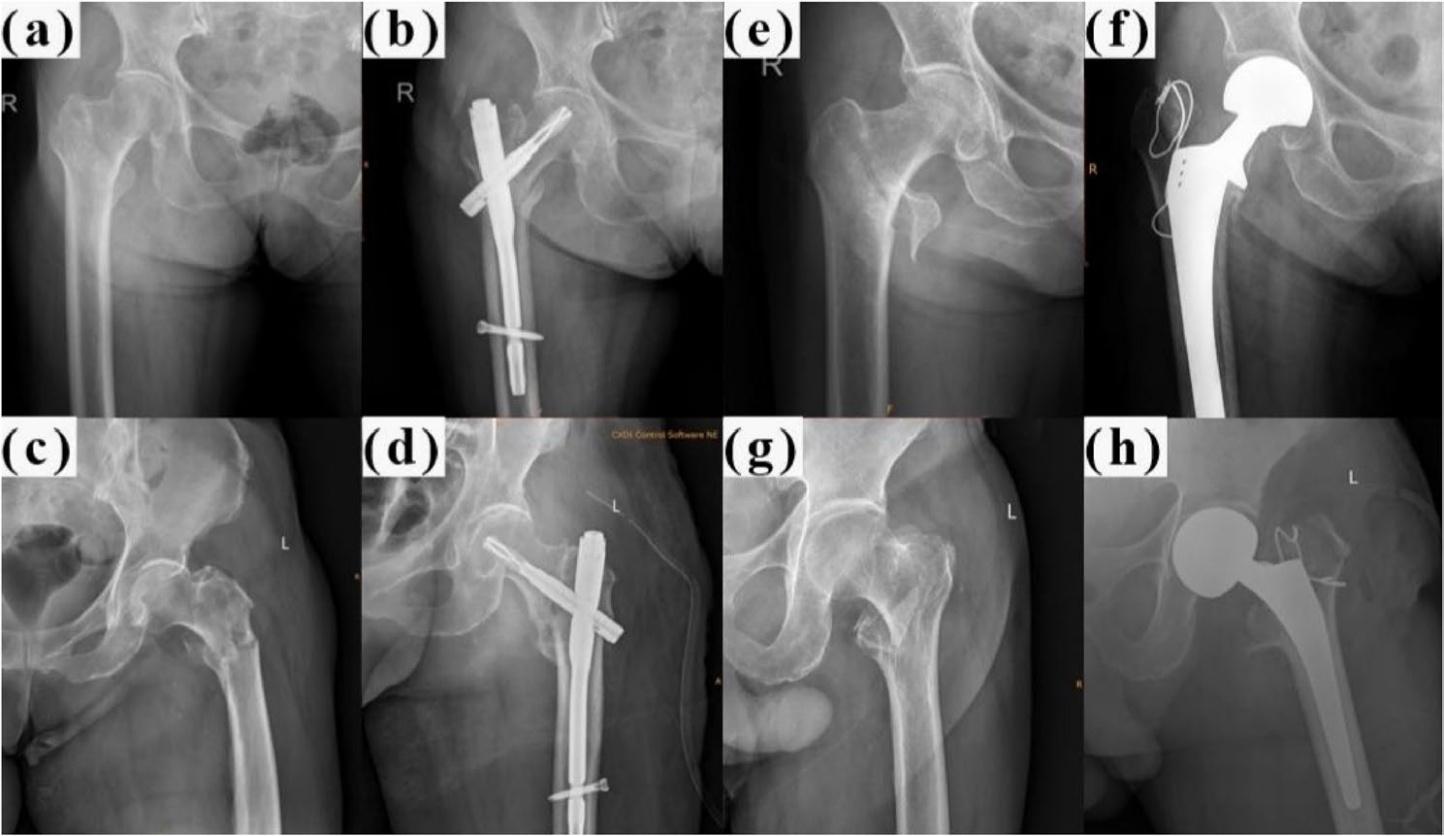
Preoperative and postoperative radiographs of various AO/OTA classifications of ITF treated by PFNA and HA. **a** Preoperative radiograph of a stable ITF (A1.1). **b** Postoperative radiograph of a stable ITF (A1.1) treated with PFNA. **c** Preoperative radiograph of an unstable ITF (A3). **d** Postoperative radiograph of an unstable ITF (A3) treated with PFNA. **e** Preoperative radiograph of a stable ITF (A1.3). **f** Postoperative radiograph of a stable ITF (A1.3) treated with HA. **g** Preoperative radiograph of an unstable ITF (A2.3). **h** Postoperative radiograph of an unstable ITF (A2.3) treated with HA. AO/OTA: AO Foundation/Orthopedic Trauma Association classification system; ITF: intertrochanteric fracture; PFNA: proximal femoral nail antirotation; HA: hemiarthroplasty

This retrospective cohort study was reported following the Strengthening the Reporting of Observational Studies in Epidemiology (STROBE) guidelines. We reviewed the records of all elderly (aged ≥ 65 years) patients with ITF admitted to our Level I trauma center between January 1, 2016, and December 31, 2022. The patient selection process is depicted in a flowchart (Fig. 1). Fractures were classified according to the AO Foundation/Orthopedic Trauma Association (AO/OTA) classification system (2018 version) [9] by two independent orthopedic surgeons. Preoperative and postoperative radiographs are shown in Fig. 2.

The inclusion criteria were as follows: (1) age ≥ 65 years [10]; (2) a radiographically confirmed diagnosis of ITF; (3) injury sustained from low-energy trauma (e.g., fall from standing height); and (4) definitive treatment with either PFNA or HA within 3 weeks of injury.

The exclusion criteria were as follows: (1) incomplete imaging or clinical data (*n* = 47); (2) medical contraindications to surgery (*n* = 11); (3) pathological fractures or high-energy trauma; (4) history of prior surgery on the ipsilateral hip (*n* = 32); (5) significant hip dysplasia (*n* = 12); (6) multiple fractures (*n* = 6); (7) preinjury ambulatory disability (*n* = 2); and (8) loss to follow-up or death from causes unrelated to the surgical intervention before the 1-year follow-up (*n* = 30).

This study protocol was approved by the authors’ Institutional Review Board (IRB). The need for informed consent was waived by the IRB due to the retrospective design of the analysis.

### Surgical indication and group allocation

Since this was a retrospective study, the choice between PFNA and HA was not randomized but determined by the senior attending surgeon. Generally, PFNA was preferred for patients with satisfactory bone stock and higher functional demands. HA was primarily reserved for patients with severe osteoporosis, significant comminution rendering anatomical reduction difficult, or those requiring immediate full weight-bearing to minimize bed-rest complications.

### Surgical procedure

The operations were performed under general anesthesia, neuraxial anesthesia, or nerve block. Tranexamic acid (0.5 g) was administered intravenously 30 min prior to the initiation of the surgical procedure [11].

- HA group

With the patient in the lateral decubitus position under general or neuraxial anesthesia, a standard posterolateral approach was employed. A posterior capsulotomy was performed, followed by a femoral neck osteotomy and extraction of the fractured femoral head. The femoral canal was prepared by sequential broaching. After trial reduction to confirm joint stability, limb length, and offset, the definitive femoral prosthesis was implanted using either cemented or cementless fixation, on the basis of bone quality and surgeon preference, and the joint was reduced [12]. The posterior capsule and short external rotators were repaired when feasible. The wound was closed in layers, with a drain placed at the surgeon’s discretion, and standard postoperative management was initiated.

- PFNA group

The patient was positioned supine on a fracture table with the affected leg slightly abducted and externally rotated. After satisfactory reduction of the ITFs, a 5–7 cm incision was made over the greater trochanter of the femur, and the soft tissues were carefully dissected to expose the bone. A guide wire was advanced into the femoral head under fluoroscopic guidance, and the helical blade was inserted over the guide wire to achieve optimal center–center positioning. The nail was subsequently locked with distal locking screws. After thorough irrigation and hemostasis, the wound was closed in layers. Postoperative fracture reduction quality was assessed using the Baumgaertner criteria, and the position of the helical blade was evaluated using the Tip-Apex Distance (TAD) to ensure optimal fixation stability.

- Postoperative treatment

Blood pressure, pulse, respiration, heart rate, and blood oxygen saturation were closely monitored throughout the entire surgery and 24 h after surgery. All patients received comprehensive postoperative care, including antibiotics, anticoagulants, analgesic agents, and incision care, according to the basic protocol.

### Outcome measures

The primary outcome measure was the Harris hip score (HHS, a validated score assessing pain, function, range of motion, and deformity), which was assessed at 12 months post-operatively. The secondary outcomes included HHS at 6 months post-operatively, total surgical duration (including anesthesia and reduction), intraoperative blood loss, total incision length, volume of blood transfused, time to postoperative weight-bearing, length of hospital stay, medical costs, mortality rates and incidence of complications (e.g., infection, deep vein thrombosis, prosthesis dislocation, and pressure ulcers) in each group. These outcomes were used to evaluate short- and mid-term effectiveness and safety.

The assessment of fracture stability was based on the AO/OTA (2018) fracture classification, specifically stratifying fractures by the integrity of the lateral femoral wall. All Type A1 fractures (A1.1–A1.3) were classified as stable, characterized by simple pertrochanteric patterns with an intact lateral wall (thickness > 20.5 mm). In contrast, fractures were classified as unstable if they exhibited lateral wall incompetence (thickness ≤ 20.5 mm), as seen in Type A2 fractures, or presented with reverse obliquity geometry (Type A3) [9]. To further elucidate potential variations in outcomes, subgroup analyses were performed within the study.

Demographic data were meticulously extracted from both paper and electronic medical records and included variables such as sex, age at the time of fracture, interval from injury to surgical intervention, fracture location, comorbid conditions, fracture classification, preoperative hemoglobin and prealbumin levels, and type of anesthesia administered.

### Statistics

Data analysis was conducted using SPSS software, version 27.0 (IBM Corp, USA). The normality of the distribution of continuous variables was ascertained through the Kolmogorov‒Smirnov test. Variables adhering to a normal distribution are reported as the mean ± standard deviation (SD) and were compared using an independent samples t test. Conversely, continuous variables exhibiting nonnormal distributions were described as medians (interquartile ranges) and were compared using the Mann‒Whitney U test. Categorical variables are presented as counts and percentages and were assessed using the chi-square test or Fisher’s exact test, as appropriate. *P* < 0.05 was considered statistically significant.

## Results

### Demographic information

Between January 2016 and December 2022, our center admitted a cohort of 497 ITF patients who fulfilled the predefined inclusion criteria. The application of the exclusion criteria resulted in the exclusion of 125 cases. Consequently, the study sample comprised 372 patients, with 153 patients in the HA group and 219 patients in the PFNA group (Fig. 1).

A comparison of the baseline characteristics between the PFNA and HA groups revealed that the two cohorts were remarkably well balanced in terms of most key clinical and demographic variables (Table 1). No significant intergroup disparities were noted in terms of sex distribution, mean age, time from injury to surgery, prevalence of comorbidities, or, most importantly, fracture classification (stable vs. unstable, *p* = 0.921). Crucially, regarding functional baseline and health status, there were no statistically significant differences in the pre-fracture mobility (independent vs. assisted, *p* = 0.598) or the Charlson Comorbidity Index (CCI) scores between the two groups. This baseline similarity in fracture stability, a primary determinant of surgical complexity and outcome, strengthens the validity of our comparative analysis. However, a statistically significant difference was observed in the type of anesthesia administered (*p* < 0.001), with nerve blocks being more prevalent in the HA group. Preoperative levels of laboratory parameters were equivalent between the two groups (Table 2).

**Table 1:** Patient demographic information

Characteristics	PFNA (*n* = 219)	HA (*n* = 153)	t/χ^2^/Z	*P*
Basic information				
Gender (N of the male)	87 (40.3%)	61 (39.9%)	0.001^3^	0.978
Age at fracture (years)	81.2 ± 7.4	82.8 ± 6.9	-1.911^1^	0.057
Time from injury to surgery (h)	97 (77.5, 129)	88.5 (71, 113.5)	-1.910^2^	0.056
Fracture site (N of left-sided fractures)	113 (51.6%)	77 (50.3%)	0.011^3^	0.917
Charlson Comorbidity Index(N)	5.4 ± 1.4	5.6 ± 1.6	-0.780^1^	0.439
Pre-fracture Mobility				
Independent	167 (76.3%)	113 (73.9%)	0.279^3^	0.598
Assisted	52 (23.7%)	40 (26.1%)		
Fracture classification (AO/OTA Fracture Classification)				
Stable	87 (39.7%)	60 (39.2%)	0.010^3^	0.921
Unstable	132 (60.3%)	93 (60.8%)		
Anesthesia				
General	34 (15.5%)	12 (7.8%)	27.262^3^	<0.001
Neuraxial	51 (23.3%)	10 (6.6%)		
Nerve block	134 (61.2%)	131 (85.6%)		

*PFNA* Proximal femoral nail antirotation, *HA* Hemiarthroplasty, *AO* Arbeitsgemeinschaft für Osteosynthesefragen, *OTA* Orthopedic Trauma Association

^1^T-test; ^2^Mann‒Whitney U test; ^3^Chi‒square test

**Table 2:** Distribution of preoperative laboratory parameters in ITF patients

Characteristics	PFNA (*n* = 219)	HA (*n* = 153)	t/χ^2^/Z	*P*
Hemoglobin (g/L)	104.1 ± 19.3	101.4 ± 17.8	1.794^1^	0.073
Platelet (10^9/L)	178.7 ± 78.2	189.8 ± 77.3	-1.808^1^	0.071
White blood cell (10^9/L)	8.8 ± 3.2	8.6 ± 3.2	0.810^1^	0.418
PT (s)	11.7 ± 1.4	11.6 ± 1.2	1.278^1^	0.202
APTT (s)	29.2 ± 3.6	29.1 ± 6.1	0.202^1^	0.840
Serum Potassium (mmol/L)	4.0 ± 0.5	4.0 ± 0.6	0.121^1^	0.903
Creatinine (mg/dL)	64.4 (52.6, 80.7)	64.3 (50.6, 84.8)	0.051^2^	0.959
AST (U/L)	22.7 (18.8, 28.4)	22.5 (18.2, 30.4)	-0.034^2^	0.973
ALT (U/L)	15.6 (11.8, 22.1)	14.0 (10.4, 23.1)	-1.280^2^	0.201
C-reactive protein (mg/L)	29.1 (9.6, 60.5)	37.8(14.1, 65.4)	1.807^2^	0.071

*ITF* Intertrochanteric fracture, *PFNA* Proximal femoral nail antirotation, *HA* Hemiarthroplasty, *PT* Prothrombin time, *APTT* Activated partial thromboplastin time, *AST* Aspartate aminotransferase, *ALT* Alanine aminotransferase

^1^T test; ^2^Mann‒Whitney U test

### Perioperative and follow-up outcomes

Surgical status was also evaluated, revealing that within the cohort, the PFNA group had a longer operative duration than the HA group, but the intraoperative blood loss was lower, the total incision length was shorter, and the blood transfusion volume was lower (*p* < 0.05) (Table 3). Compared with those in the HA group, patients in the PFNA group had longer postoperative weight-bearing times and hospital stays, but the hospitalization costs were significantly lower (*p* < 0.05).

**Table 3:** Perioperative and follow-up outcomes between the PFNA and HA groups

Characteristics	PFNA (*n* = 219)	HA (*n* = 153)	t/χ^2^/Z	*P*
Primary outcome				
HHS 12 months after surgery	87.9 ± 5.8	84.6 ± 6.3	4.522^1^	<0.001
Secondary outcomes				
HHS 6 months after surgery	67.6 ± 9.2	68.6 ± 8.5	-0.955^1^	0.340
Operative duration (min)	129.5 ± 53.2	116.5 ± 42.7	2.239^1^	0.026
Intraoperative blood loss (ml)	185.3 ± 125.5	333.2 ± 132.5	-9.347^1^	<0.001
Total incision length (cm)	7.1 ± 2.6	14.5 ± 2.8	-23.197^1^	<0.001
Blood transfusion volume (ml)	0 (0, 400)	400 (300, 400)	4.490^2^	<0.001
Postoperative weight-bearing time (days)	8.5 ± 3.7	4.5 ± 3.3	7.970^1^	<0.001
Hospital stay (days)	13.7 ± 4.4	11.8 ± 3.8	3.179^1^	0.002
Medical cost (10^3^ dollars)	4.8 (4.2, 6.4)	7.2 (5.4, 8.9)	6.284^2^	<0.001
Mortality (%)	7 (3.2%)	9 (5.9%)	1.579^3^	0.209
Complications				
Pulmonary disease	2	2	-	1.000^4^
Cardiovascular disease	8	3	-	0.536^4^
Thrombogenesis	1	1	-	1.000^4^
Gastrointestinal bleeding	2	0	-	0.514^4^
Urological disease	1	1	-	1.000^4^
Total	14 (6.4%)	7 (4.6%)	0.559^3^	0.455

*PFNA* Proximal femoral nail antirotation, *HA* Hemiarthroplasty, *HHS* Harris hip score

^1^T-test; ^2^Mann‒Whitney U test; ^3^Chi‒square test; -, not applicable; ^4^Fisher’s exact test

Compared with those in the HA group, patients in the PFNA group had higher HHSs 12 months after surgery (87.9 ± 5.8 vs. 84.6 ± 6.3, *p* < 0.001). There was no significant difference in HHS between the two groups at 6 months after surgery (67.6 ± 9.2 vs. 68.6 ± 8.5, *p* = 0.340). The mortality (3.2% vs. 5.9%) and complication (6.4% vs. 4.6%) rates of the PFNA and HA groups were not significantly different.

### Complications

Regarding complications, 14 adverse events (6.4%) were recorded in the PFNA group, including cardiovascular events (*n* = 8), pulmonary issues (*n* = 2), gastrointestinal bleeding (*n* = 2), a thrombotic event (*n* = 1), and a urological issue (*n* = 1). In the HA group, 7 events (4.6%) were recorded, including cardiovascular events (*n* = 3), pulmonary issues (*n* = 2), a thrombotic event (*n* = 1), and a urological issue (*n* = 1). The difference in the overall complication rate between the groups was not statistically significant (*p* = 0.455).

### Subgroup analysis

Stratifying the analysis by fracture stability provided critical insights (Table 4). In patients with stable ITF, there was no significant difference in the 12-month HHS between the PFNA and HA groups (87.5 ± 4.6 vs. 88.3 ± 4.4, *p* = 0.330). In patients with unstable ITF, a clear temporal divergence in recovery emerged. The HA group demonstrated a marginal functional advantage at 6 months post-operatively (HHS: 70.4 ± 8.2 vs. 66.9 ± 10.1, *p* = 0.030), reflecting faster initial recovery due to immediate weight-bearing. However, by 12 months, the PFNA group achieved a significantly greater HHS (88.3 ± 6.3 vs. 82.5 ± 6.3, *p* < 0.001).

**Table 4:** Comparison between stable and unstable ITF subgroups

	Stable ITF			Unstable ITF		
Characteristics	PFNA (*n* = 87)	HA (*n* = 60)	P	PFNA (*n* = 132)	HA (*n* = 93)	P
Gender (N of the male)^3^	18 (34.6%)	16 (29.6%)	0.628	69 (41.3%)	45 (45.5%)	0.510
Age at fracture (years)^1^	80.4 ± 6.4	82.3 ± 6.8	0.069	81.7 ± 9.7	83.1 ± 7.0	0.051
Time from injury to surgery (h)^1^	6.5 (4, 85.25)	7 (5, 14)	0.667	99 (76, 130.5)	80 (39, 105)	0.477
HHS 6 months after surgery^1^	68.6 ± 7.4	66.1 ± 8.5	0.114	66.9 ± 10.1	70.4 ± 8.2	0.030
HHS 12 months after surgery^1^	87.5 ± 4.6	88.3 ± 4.4	0.330	88.3 ± 6.3	82.5 ± 6.3	<0.001
Operative duration (min)^1^	115.6 ± 36.7	99.2 ± 28.0	0.019	130.2 ± 48.0	123.2 ± 41.2	0.289
Intraoperative blood loss (ml)^1^	172.8 ± 101.5	311.3 ± 130.7	<0.001	190.5 ± 134.3	345.0 ± 132.8	<0.001
Total incision length (cm)^1^	6.1 ± 1.4	13.9 ± 1.8	<0.001	7.6 ± 2.9	14.9 ± 3.1	<0.001
Blood transfusion volume (ml)^2^	0 (300, 400)	400 (225, 650)	0.129	0 (0, 400)	400 (300 ,400)	<0.001
Postoperative weight-bearing time (days)^1^	6.6 ± 1.9	3.6 ± 1.4	0.001	8.5 ± 3.7	5.0 ± 3.4	<0.001
Hospital stay (days)^1^	13.8 ± 3.4	10.0 ± 1.6	0.004	14.3 ± 5.8	13.6 ± 9.7	0.002
Medical cost (10^3^ dollars)^2^	4.0 (3.3, 5.0)	7.8 (6.5, 9.1)	<0.001	5.3 (4.5, 6.7)	6.4 (5.0, 8.9)	0.002

*ITF* Intertrochanteric fracture, *PFNA* Proximal femoral nail antirotation, *HA* Hemiarthroplasty, *HHS* Harris Hip Score

^1^T-test; ^2^Mann‒Whitney U test; ^3^Chi‒square test

In both the stable subgroup and the unstable subgroup, PFNA was consistently associated with less intraoperative blood loss, smaller incisions, and lower medical costs but at the expense of delayed weight-bearing (Table 4). In the unstable subgroup, this indicates a trade-off where HA prioritizes early recovery, whereas PFNA excels in long-term function.

## Discussion

The principal finding of this study is that, in treating unstable elderly intertrochanteric fractures, there is a clear clinical trade-off between PFNA and HA: HA offers advantages in terms of faster early weight-bearing, whereas PFNA demonstrates superior functional recovery at 1 year post-operatively. This discovery suggests that a universal treatment algorithm is inappropriate; instead, the choice should be individualized on the basis of the patient’s life expectancy and functional needs. This retrospective cohort study provides a nuanced comparison of PFNA and HA for treating elderly patients with intertrochanteric fractures, uniquely stratifying the analysis by fracture stability.

We must acknowledge a significant limitation and potential source of bias in our study: although baseline characteristics were well balanced, subtle selection biases may exist, such as surgeons preferentially choosing HA for frailer patients or those with more comminuted fractures deemed unsuitable for internal fixation, even if not reflected in the AO/OTA classification. Paradoxically, this bias makes our key finding even more compelling. Despite being potentially used in a cohort with more challenging cases, PFNA still achieved superior 12-month functional outcomes, strengthening the conclusion that it is a highly effective long-term solution for unstable ITF.

The superiority of PFNA in managing unstable fractures is likely attributable to its biomechanical advantages. As an intramedullary device, PFNA provides a stable, load-sharing construct that controls both varus collapse and rotational forces, which are critical challenges in unstable patterns (e.g., reverse obliquity, lateral wall comminution) [13]. By preserving the native bone stock and promoting biological healing, PFNA facilitates a more durable long-term functional outcome once fracture consolidation is achieved. In contrast, while HA offers immediate stability, its success in unstable fractures—especially in osteoporotic bone—can be compromised by risks such as subsidence, periprosthetic fracture, and dislocation, potentially leading to inferior long-term function [14]. Our findings align with a growing body of literature suggesting that for active elderly patients with unstable fractures, the long-term benefits of biological fixation may outweigh the advantages of immediate prosthetic replacement [8, 15]. It is important to note that the functional superiority of PFNA relies heavily on surgical quality. In our cohort, strict adherence to surgical principles, such as achieving a TAD < 25 mm and acceptable reduction according to Baumgaertner criteria, was emphasized to minimize the risk of mechanical failure.

A noteworthy finding was the temporal divergence in functional outcomes. While no significant difference in HHS was observed at 6 months, the advantage of PFNA became evident at 12 months. This pattern reflects the fundamental trade-off between the two techniques. The long-term superiority of PFNA likely stems from preserving the native joint and providing better biomechanical reconstruction [13, 14], whereas the early benefits of HA arise from immediate joint stability without the need for fracture healing [8, 12]. This trade-off underscores the need for personalized approaches. HA, as a primary arthroplasty, permits immediate, full weight-bearing, which likely facilitates faster initial recovery and explains the comparable 6-month scores [16]. This early mobilization is a key advantage, potentially reducing complications such as pneumonia and thrombosis associated with prolonged recumbency [17]. Conversely, PFNA requires a period of protected weight-bearing to allow for fracture healing. The superior 12-month HHS in the PFNA group suggests that once biological union is complete, the reconstructed native hip provides better overall function than a prosthesis. This finding is corroborated by studies indicating that while hemiarthroplasty patients may initially show better activity, those treated with intramedullary fixation often catch up and eventually surpass them in long-term functional capacity [12, 18].

Our analysis of perioperative outcomes further illuminates the distinct profiles of each procedure. PFNA is minimally invasive, with significantly less intraoperative blood loss, smaller incision lengths, and lower medical costs [16, 17, 19]. The cost-effectiveness of PFNA is likely attributable to the lower expense of implants than prosthetic components required for HA [20, 21]. Except in cases where lateral wall damage necessitates extended incisions and plate-screw fixation [22], PFNA causes less muscle tissue damage, which is particularly beneficial for frail elderly patients for whom extensive surgery and blood loss cause considerable physiological stress. The observation that PFNA had a longer operative duration in our cohort, which contrasts with the findings of some previous studies [15, 19], is critically important to interpret. We attribute this discrepancy to our inclusion of fracture reduction time within the total operative duration. Achieving an acceptable, often closed, reduction for an unstable fracture can be technically demanding and time-consuming, but it is a prerequisite for successful PFNA fixation, not a component of HA surgery.

Synthesizing our findings, the choice between PFNA and HA should be tailored on the basis of fracture stability, patient physiology, and long-term expectations. For stable intertrochanteric fractures, both techniques are viable options, and the decision can be guided by factors such as surgeon preference and institutional protocols. However, for elderly patients with unstable intertrochanteric fractures, our evidence suggests a clearer path. PFNA may be preferred for relatively active patients with reasonable bone quality and longer life expectancy, as it offers the prospect of superior long-term hip function, despite a slower initial recovery. Conversely, for a frailer patient with severe osteoporosis, significant comorbidities, or limited life expectancy, HA remains an excellent option, prioritizing rapid mobilization and minimizing the risks of fixation failure and prolonged bed rest. This study reinforces the need for a personalized approach, moving beyond a “one-size-fits-all” paradigm.

### Limitations

We acknowledge several limitations. First, as a retrospective study, it is susceptible to selection bias and unmeasured confounders. The most significant difference was the difference in anesthesia type between the groups. This may reflect underlying differences in patient frailty or surgeon preference that were not captured in our data. While regional anesthesia is often preferred for more comorbid patients, its direct long-term effect on functional outcomes remains controversial. Second, we excluded patients lost to follow-up or those who died within one year. We acknowledge that this may introduce survivorship bias, as the excluded patients might represent a frailer subset with poorer functional potential. However, given that mortality rates were similar between groups (3.2% vs. 5.9%, *p* = 0.209), this bias likely affects both cohorts equally. Third, although the AO/OTA classification is standard, we did not quantify bone mineral density, which could influence outcomes. Fourth, the procedures were performed by two distinct surgical teams (trauma and arthroplasty), introducing potential variability in technique and perioperative protocols. Fifth, the one-year follow-up is insufficient to assess long-term outcomes such as implant survivorship and revision rates. Finally, our use of HHS is subject to known ceiling effects, which may limit its ability to detect subtle functional differences in high-performing patients. Future multicenter, prospective, and ideally randomized controlled trials that incorporate propensity score matching and patient-reported outcomes beyond HHS are needed to validate our findings.

## Conclusion

For elderly patients with stable ITF, there was no significant difference in clinical outcomes between PFNA and HA. However, among those with unstable ITF, a clinical trade-off exists: HA provides faster early mobilization, whereas PFNA offers superior long-term hip function at 12 months post-operatively. These findings suggest that implant choice should be tailored to patient life expectancy and functional goals, with PFNA potentially more suitable for those prioritizing long-term outcomes, while HA remains a robust choice for patients with limited physiological reserve.

## Data Availability

The datasets generated and/or analyzed during the current study are not publicly available owing to privacy and ethical restrictions related to patient information but are available from the corresponding author upon reasonable request.
